# “Antisocial Today and Also Always”?: A Qualitative Examination of Engineering Students’ Social Considerations in Robot Design for Healthcare

**DOI:** 10.1007/s11948-026-00610-2

**Published:** 2026-07-24

**Authors:** Sachil Singh, Amy R. Wu

**Affiliations:** 1 Faculty of Health, York University, Toronto, Ontario Canada; 2 Mechanical and Materials Engineering, Ingenuity Labs Research Institute, Queen’s University, Kingston, Ontario Canada

**Keywords:** Algorithms, Bias, Blind spots, Engineering education, Ethics, Healthcare, Robot design

## Abstract

In this article, we investigate influences of social positionality, designer bias and educational exposure on algorithmic design in healthcare. Against the backdrop of literature on designer bias that points to how it contributes to disproportionate, discriminatory and unethical impacts for racialized and gendered bodies, this study tests this argument with an experiential case study involving Aldebaran’s NAO robot and mechatronics and robotics engineering students at a university in S. E. Ontario, Canada. To conduct the study, we adopted open-ended and semi-structured interviews. The study sought to address questions such as: what other social agents – besides engineers – are/should be involved in design? To what extent do engineers collaborate with other knowledge holders? From students’ point of view, how does engineering education prepare them to tackle ethical and social problems in design? Because of the small sample size, the intention is not to extrapolate beyond the data in this study but for the data to serve exploratory purposes in pursuit of further research.

## Introduction

Safiya Noble’s (2018) breakthrough book *Algorithms of Oppression*, brought attention to algorithms as social constructs that are not the objective technologies they are often portrayed to be. The book provides a powerful exploration of the racial and gendered consequences of Google searches, which, in turn, reveal the subjectivities of designers. She argues that, “*If Google software engineers are not responsible for the design of their algorithms, then who is?*” (Noble, 2018, p. 66, emphasis added). On the one hand, this argument can be broadened to a logical connection between design and engineers by holding them fully accountable for discriminatory outcomes. Such framings provide the research problem that we tackle in this article: against the backdrop of literature on designer bias that points to how it contributes to disproportionate, discriminatory and unethical impacts for racialized and gendered bodies, this study tests this argument with an experiential case study involving Aldebaran’s NAO robot and mechatronics and robotics engineering students at a university in S. E. Ontario, Canada. The research questions that we pose are: which other social agents – besides engineers – are/should be involved in design? To what extent do engineers collaborate with other knowledge holders? From students’ points of view, how does engineering education prepare them to tackle ethical and social problems in design?

The article proceeds with five main sections. Firstly, “What is NAO?” briefly introduces the social robot that is used in this study. Secondly, the “Methods” provide the rationale and strategy for addressing the research questions, while the third section presents the “Findings” from our fieldwork. Fourthly , “Discussion” of our findings is provided, followed by our “Conclusion” which includes insights for further research.

In light of concerns similar to those that Noble raises above, there has been a strong push to infuse current engineering education with social and ethical responsibility (American Society for Engineering Education, 1999; Engineers Canada, 2023; Han, 2023; Hollander & Kahl, 2010). While there can be multiple facets to and interpretations of responsible engineering (e.g. social relevance (Lim et al., 2021), safety (Hayes et al., 2025), risk mitigation (Wyndham et al., 2015), inclusive design (Criado-Perez, 2019), sustainability (Saari et al., 2024)), it typically manifests in engineering education through “engineering ethics” which considers public welfare paramount (Engineers Canada, 2023). Motivated by infamous historical disasters, such as the 1906 Quebec Bridge Collapse and the 1940 Tacoma Narrows Bridge Collapse, engineering ethics aims to educate future engineers on how poor engineering design and practices can be detrimental to the public (Thilmany, 2023). In some countries, professional engineering organizations have mandated the inclusion of engineering ethics for the regulation of the engineering profession. For example, within Canada, one cannot formally practice engineering without an engineering license (Engineers Canada, n.d.). In addition, engineering professions expect all members to uphold a Code of Ethics which include highest regard for public welfare safety, maintaining professional integrity, and performing engineering tasks only within areas of competence (National Society of Professional Engineers, 2019; Ontario, 2025). In addition, the topic of engineering ethics must be included for accreditation of some undergraduate engineering programs (ABET, n.d.; Engineers Canada, 2020), mandating that students should be able to “produce solutions … with consideration of public health, safety, and welfare …” (ABET, n.d.). While engineering ethics teaches students to be responsible for the safety (e.g. its structural integrity) and accountability of their design, it does not necessarily explicitly address other areas such as inclusivity and bias, which could detrimentally affect certain populations (Criado-Perez, 2019; Noble, 2018).

Despite the emphasis on engineering ethics in education, previous studies have found a lack of targeted focus on ethical training as a foundational engineering topic (Barry & Ohland, 2012; Hess & Fore, 2018). Even the implementation of engineering ethics in education has been pedagogically challenging with mixed and inconsistent approaches across universities (Hess & Fore, 2018; Martin et al., 2021b; Sunderland, 2019). Hence, it is perhaps unsurprising that its effect in engineering education has been varied and unclear (Howland et al., 2024). For example, studies utilizing the Defining Issues Test, Version 2 (DIT-2) (Rest et al., 1999) to measure moral judgement have found no changes (Drake et al., 2005; Wu et al., 2008) to moderate increases in DIT-2 scores (Loui, 2005) after some exposure to ethics in engineering education.

Howland et al. (2024) employed three different surveys to investigate undergraduate engineering students across multiple years in four different universities. Broadly, they found increased knowledge of ethics and consistent levels of “social consciousness” over time (Howland et al., 2024), in contrast to previous work that found decreased concern for public welfare (Cech, 2014). The latter work and others argued that a ‘culture of disengagement’ was pervasive in undergraduate engineering education because engineering culture devalued non-technical knowledge and social competencies (Cech, 2014; Martin et al., 2021a; Niles et al., 2020). In Lim et al.’s study, they found that many students were unaware, uninterested, or unwilling to discuss the broader ethical and social implications of their course materials, which highlights some of the difficulties behind implementing effective engineering ethics education (Lim et al., 2021).

These findings may be alarming as technology, fueled by advances in robotics and artificial intelligence, are increasingly prevalent in our lives. Within healthcare, robots are starting to be used as greeters (Humber River Health, 2018; Øllgaard et al., 2025; Saad, 2025) and for delivery of medication and supplies (Johnson, 2022). They are also used to relieve nurse workload by autonomously taking blood samples from patients (Wang et al., 2025). These technological advances are fraught with social and ethical concerns that need to be addressed for safe and effective implementation (Kaliya-Perumal & Sidarta, 2025; Rasmussen et al., 2024). To empirically explore the contribution of engineers to disproportionate, discriminatory and unethical impacts of design for racialized and gendered bodies, we designed a mock programming exercise at a university in S.E Ontario. This exercise was designed with a commonly used social robot that has potentially real implementation in the medical field.

## What is NAO?

**Fig. 1:**
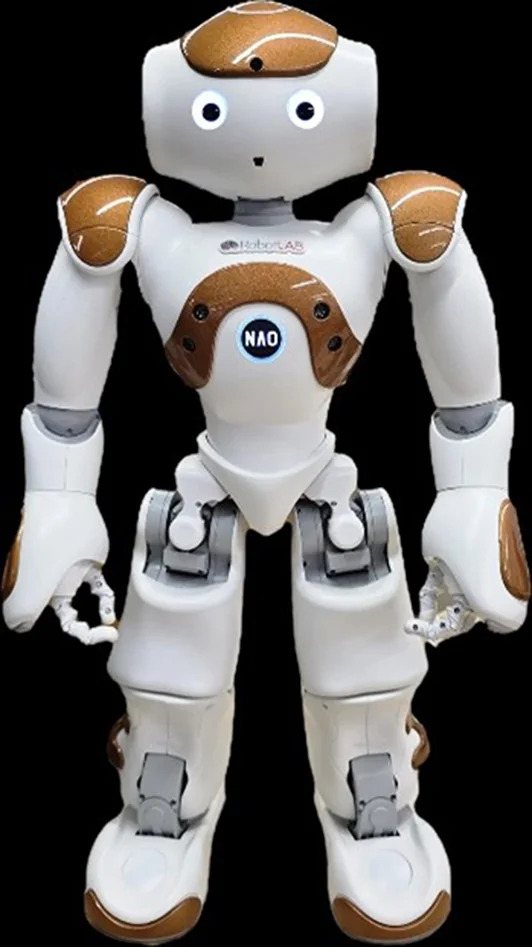
NAO robot (personal image)

Manufactured in France by Aldebaran (founded in 2005), NAO (pronounced *now*) is an autonomous humanoid robot that stands at 58 centimetres tall and weighs 5.5 kilograms (see Fig. 1). The robot is fully programmable and equipped with microphones and loudspeakers for speech-based interaction (Aldebaran, 2025). The subject areas of deployment range from a robot soccer league to the fields of education and health as a “socially assistive robot” or “social robot”. Some of the intended benefits of NAO are in the healthcare and educational sectors. In healthcare settings, NAO assists with patients’ physical rehabilitation, acts as a mediation tool during autism therapy, and provides emotional behaviour to mimic social presence (Maxtronics, 2026a). NAO can also be utilized as a programming tool to develop programming and robotics skillsets in schools (Maxtronics, 2026b). NAO’s child-like size and appearance make it appealing for robot-assisted interventions or therapy for children (Cabibihan et al., 2013; Robins et al., 2006). For example, NAO has been shown to improve attention of children with autism (Tapus et al., 2012), enhance social and cognitive skills of preschool children (Feidakis et al., 2025), and increase engagement and critical thinking for students across different educational levels (Chalmers et al., 2022). It is therefore evident that NAO has a clear social purpose. The version used for the study was the NAO V6 AI Edition (Aldebaran, Paris, France).

We chose NAO for our study due to its promise and popularity in health-related research, coupled at the same time with a dearth of social analysis. While numerous social robots or socially assistive robots exist, the NAO robot is commercially available and has shown potential in clinical settings across a wide range of applications that necessitate social considerations. This particular robot, or a similar robot, is a likely platform that our future roboticists will encounter and need to program. In addition, NAO’s ease of programming, without the need to learn a new language or understand existing coding languages, makes the platform more accessible for investigations with students of varying programming abilities and backgrounds.

## Methods

Because this is exploratory research that does not project findings beyond the scope of this study, we do not seek objectivity through empirical positivism but rather find richness in participants’ subjectivities. We are interested in the mutual constitution of observable reality and the words used to describe that reality. This invites the participation of non-human actors into the research (such as NAO, objects that participants describe in the room, participants’ chosen clothing etc.) as much as human actors (Latour, 1999). This study borrows from qualitative research methods to address the primary research aim and research questions that were presented in the Introduction. With the use of experiential learning and open-ended semi-structured interviews, undergraduate student participants were asked to code the NAO robot in response to a prescribed health-oriented task and then reflect on their experiences. It is recognized in qualitative research that such methods are most effective “to establish the flow of inquiry” and observe “the depth of the participants’ experiences” (Maissa, 2014). In previous work, we have had success with open-ended and semi-structured interviews, in particular, as a method to build rapport with participants and provide them with opportunities to tell their own stories (Singh, 2023).

To better understand the robot from a research point of view, an OMNI database search – an academic search tool for Ontario, Canada university libraries – was conducted for all peer-reviewed articles containing the words “NAO robot” between 2001 and 2026, receiving a total of 539 results. However, only 46 of these results were in the area of social science, indicating that despite the robot’s popularity, social analysis is lacking and under researched. In fact, one of the articles in the search claims that “[a] humanoid robot’s development requires an incredible combination of interdisciplinary work from engineering to mathematics, software, and machine learning” (Baothman, 2021, p. 1). Despite NAO being designed to interact with people, and research studies that have shown NAO’s promise as a socially assistive robot, the absence of sociological literature is striking. In fact, Aldebaran’s description of the robot is that it is designed for “Education and Healthcare” but the absence of a sociological lens implies that there is limited work on common social problems such as designer bias, designer positionality, inclusive design, and social impacts on users.

Ethical clearance was obtained from two universities in the last quarter of 2024 and recruitment occurred at a university in S.E. Ontario, from January to October 2025. In total, 5 students (*n* = 5) responded to the recruitment methods of email advertisements on departmental listservs, collegial promotion of the study in undergraduate courses, and participant snowballing. All participants are pseudonymized in this study and were provided with a 15CAD coffeeshop gift card for their time. Despite our active recruitment strategies, response rates remained consistently low. In the context of rapidly evolving technologies in mechatronics and robotics, there was a need to conclude the research after one year and seek publication while the study still had relevance for readers from different disciplines. Although the small sample size by the end of this study period does not permit projections to a broader population, the insights gleaned from the in-depth interviews satisfy the exploratory nature of this study.

During the study, each participant was invited to a private room where an undergraduate research assistant provided training on how to use the robot software (15 to 30 minutes). This was followed by a testing phase in which the participant was afforded the opportunity to familiarize themselves with the robot software (15 minutes). They were then given time to design the code for a pre-determined scenario in which the NAO robot had to be programmed to write prescriptions for patients (60 minutes; see Appendix A).

The scenario that was provided to respondents was futuristic and controversial, with intentional ‘red flags’ embedded in the process. These red flags were derived from social science literature as key concerns of a ‘technological turn’ that underscore existing social inequalities of race and gender in particular (eg: Benjamin, 2019; Browne, 2015; Noble, 2018). The red flags identified in this study included: autonomous robot capability in lieu of trained healthcare professionals; the ability of the robot to engage with the patient sufficiently to gather enough information to write prescriptions; the need for patient race data to inform the prescription; and the collection of participant self-identifying data (age, race and gender) in the interview.

These red flags were included – but not framed as such – to assess respondents’ levels of comfort with them and to observe if any opposition would be expressed to their appropriateness. This was important for the study because it allowed the research team to observe participants’ (ie. engineers-in-training) willingness to ask questions about the robot design, their confidence to refuse participation in aspects of the study, and their views on the ethics of robot design in healthcare.

Following the 60-minute programming session, we conducted a 20–30 minute interview that reflected on the participant’s learnings, troubles and experiences. The reason that an open-ended approach was adopted for the study is because it provides necessary flexibility to guide the conversation based on what a participant chooses to focus on or emphasize. At the same time, a semi-structured approach provides sufficient consistency in the line of questioning for the purposes of comparison in the analysis. The interviews were recorded with a designated recording device, transcribed by a third-party professional transcription service, and then coded and analysed by the research team. This process involved both content analysis and discourse analysis to identify recurring themes and to compare participants’ descriptions of experiences during the scenario.

The interview sought to understand participants’ experiences with NAO within the context of robots playing greater roles in patient care. Besides asking students about their experiences with programming NAO, the interview sought to get at ethical questions pertaining to the role of the designer.

## Findings

### Participant Background

To gain some understanding of participants’ social backgrounds, they were asked for their self-identification, as well as the reasons that led them to pursue their current studies (Table 1). Against the backdrop of literature that comments on how white men largely inform disproportionate outcomes in the field of engineering (eg: Criado-Perez, 2019; Noble, 2018), it was important to understand participants’ social positionalities. All participants ranged in age from 19 to 21. Two self-identified as white women and three self-identified as men. While some participants described their interest in computer engineering and robotics in terms such as “robots are quite cool” (Sanath), two participants drew on personal experiences that shaped their trajectories. Chloé reported that attending a workshop for women in Science, Technology, Engineering, and Mathematics (STEM) motivated her to pursue her field. Her eyes widened as she commented, “I thought all the electrical engineering stuff was really cool and so I started applying to engineering programs.” As a Fourth Year 21-year old, Chloé has ambitions to change the face of computer engineering from what she sees as predominantly male. Similarly, Rebecca was inspired by an engineering workshop for women but also by her father who is in the same general field.

**Table 1:** Participants’ self-identification and program of study

Participant Name (pseudonymized)	Self-identification	Program of Study
Chloé	“I am 21 years old. I identify as a woman, and I am white.”	Mechatronics and Robotics Engineering Program
Brandon	“… man, or male. My mother is from Scotland heritage, my father’s from Mexican heritage if that is what you mean … that’s my background. I’m here; I’m Canadian.”	Mechatronics and Robotics Engineering Program
Michael	“I’m 21 years old, and I’m a male, man, and my race, ethnicity would be Caucasian, European.”	Computer Engineering
Sanath	“I am … Bengali, Bangladeshi, so I guess that would be brown, Southeast Asian, dark beige.”	Mechatronics and Robotics Engineering Program
Rebecca	“I am 21 and I am a white woman.”	Applied Mathematics and Engineering Program

### Responsibility in Design

Against the backdrop of the view that many engineers lack social responsibility in design, the interview sought to unpack participants’ social postures and ethical drives on such matters. Chloé described how training robots on limited accents not only diminishes a robot’s ability to understand someone’s speech but reflects designer subjectivities. When discussing healthcare, she noted that “there’s a lot of flaws of studies based on old white men, where they do research based on them, and they’re like, ‘This is how you give healthcare to people.’” She also raised examples of how people are taught that someone turns blue if they can’t breathe but this overlooks how this might not be apparent for a darker skinned person. “We didn’t consider this” she adds in frustration. Her final example was that of endometriosis which is a condition that affects the uterus. “[T]he research on it is terrible … because it largely doesn’t affect men”.

For Brandon, a Fourth Year male of Scottish/Mexican heritage, focus was more on individual responsibility than the systemic concerns raised by Chloé: “Well, I think as an engineer, you are responsible for the work that you do”. Similarly, Michael said that they would stand up for their beliefs if they encountered something unethical in design because “I want to do the right thing … otherwise I don’t have a purpose to my life, honestly.” There were also views amongst participants that of all those involved in design, engineers should be the most accountable for their work. Chloé stated, “So I think that the engineer probably bears the brunt of the responsibility, because they are the person who, essentially, at any time, has the power to just pull a switch and be like, ‘I’m not, in fact, going to program it to do this.’” This connects to Brandon who commented that “If someone assigns you a task you should use your own conscience to decide that” because a designer needs to employ critical thinking. By contrast, Rebecca was the only participant to comment on how priorities influence her sense of responsibility in design. When discussing a designer’s responsibility with the task provided, she said that her dedication to the task with NAO was limited because “I’m hoping to do some more [school]work today”. Therefore, “I didn’t put all the work into it.”

### Disciplinary Blind Spots

One of the key research questions in this study is: ‘what other social agents - besides engineers - are/should be involved in design?’ To address this, interview questions sought to examine study participants’ appreciation of their own disciplinary limits, and the extent to which they believed that multi-disciplinary collaboration is important in design. Most participants commented on the need for cross-disciplinary collaboration when it comes to design. Sanath characterized this as “safety roundtables” where design is collaborative. He explained that who is present at the table depends on the task at hand. He elaborated that “… if [robots] are meant to interact with humans, you need to have social scientists that really understand, one, how humans interact with each other, but also how humans will interact with computers and robots, if that is like a field. I’m sure that is. Because engineers themselves do not study that, we study the physics, we study the design principles, and we study some ethics …”

Similarly, Brandon sees the value in not relying solely on engineers when it comes to design:

I think that I’m a big proponent of collaboration … the more voices, the better. So, if you’re implementing a robot that’s going to be implemented in healthcare you should have as many sides to that story as possible. Like the healthcare provider who is going to use the tool, the patient who’s going to receive support, use from that tool, the engineers who know about the capabilities of what can or can’t be implemented. Law, someone in politics or sociology or something who knows about, I don’t know, behaviour and whether this is a good idea to moving forward, or what the consequences are in the future of that decision. I think that robotic tools can be a great thing, and I think it’s a slow process to get people to understand exactly what the benefits will be. And I think that that kind of communication is important, and the more people you can have present in that … the better.

In light of interview questions about disciplinary limits and the potential for cross-disciplinary collaboration, it was noticeable that Chloé wore a black t-shirt that read ‘Antisocial Today and Also Always’. Seemingly hinting at this message, she, and Rebecca, did not discuss collaboration of equal disciplinary partners like the other participants. Instead, Chloé and Rebecca phrased this collaboration as “consultation” with doctors and patients such that the engineer is the lead figure. This framing renders the engineer in control of the process which was confirmed with the statement: “But I think that to a large extent, it is your responsibility [as the engineer] what the thing you’ve created does, and so you have to be very careful to ensure that you are not doing any harm with that device.”

### Engineering Curriculum

To respond to the research question ‘Are engineers trained to be mindful of ethical and social problems?’, participants were asked to reflect on the extent to which ethics and other social considerations are present as a theme in their engineering courses. There was consistency in responses about exposure to such themes in the First Year curriculum. However, views on the inclusion of designer ethics in subsequent years of the Degree were inconsistent. Perhaps more notable were comments on the weighting of ethics components in course work. When asked if it is possible to pass a course by focusing only on the design components and ignoring the ethics modules, Sanath responded swiftly, “Yes, yes, 100%.” He went on to comment that “a lot of the engineers are like, ‘Man, just let me build. Let me do the technical stuff. Let me focus on the ethics later.’” This is linked to Michael’s observation that “… ethics comes up, but at least most people that I’ve … encountered … don’t … see it as that important”. This mindset might stem from what Sanath explained to be an absence of much ethics in his robotics program. Even if a student wishes to take ethics-focused courses, electives are not possible until Fourth Year. These concerns were shared by Chloé who pointed out that, “engineers should have a dedicated course on ethics in design and on how to design responsibly and the responsibilities of a designer, but we currently do not have a course like that.” Rebecca disagreed on the inclusion of such a course: “if there was a whole other class for ethics-based learning, I mean that would eat up a lot of my time and, yeah, I probably wouldn’t be happy about that.”

### Dealing with Red Flags

As noted above, a number of red flags were intentionally incorporated into the participant experience to observe how participants would handle them. All but one participant (Brandon) accepted the task of programming NAO to ask patients for their race. Brandon was hesitant and asked if he had to follow this instruction. It was clarified that he should do what he believed was necessary to handle the prompt. In the end, he refused to include a question about race. In the interview, he explained his hesitation with race as a variable in design: “What threw me off was that [race] might inform how much pain medication they receive … to say that because of their race, you would give them more or less pain medication, I disagree with that. I don’t think that should be part of that.” We pushed Sanath on this response with an elaboration on the hypothetical scenario: his boss – who pays his salary – has tasked him with including race in the algorithm. Sanath responded that,

if you are to do the work, then you are responsible for the consequences of that. And that should be heavy on your mind, I think. So, even though they’re paying your salary, I think it’s very important that you stand up for your ethical duties. And if that’s a complaint that needs to be brought up to, I don’t know, law enforcement in that case, then that should be what happens.

Other ethical concerns were shared by a few participants. These pertained to the need to code a prompt for the patient to opt-out. In fact, this was the first goal for three participants because, as Chloé put it, “a lot of patients may not be comfortable with having a robotic nurse”. Brandon commented that “you need to give some agency to the patient”. For Sanath, “people deserve … the right to opt out of services that might be, I guess, too new or too unfamiliar to them … you always need some type of emergency stop or some sort of way to communicate, like, “Hey, like, this is not working for me. I need somebody else.”

## Discussion

### Are Robotics Engineering Students on a Path to Being “Antisocial Today and Also Always”?

Given the investigation into robotics engineers’ interactions with other knowledge groups, Chloé’s message on her t-shirt was an interesting thinking point for the interviewer because it encapsulates a main theme of this project. On the one hand, “Antisocial Today and Also Always” aligns with popular social science literature on the shortcomings of engineers. On the other hand, Chloé’s insights in the interview render this a tongue-in-cheek message, particularly directed at those very social scientists. Indeed, our research demonstrates that there is a lot to be debunked when it comes to the stereotypical role of robotics engineers because the student participants offered many challenges to the assumptions.

Our findings suggest that statements about the roles, decisions, biases and blind spots of robotics engineers need to be context-specific. To project beyond the sample can be premature and fall short of appreciating engineers’ human-centred and inclusive design approaches. Interviews revealed participants’ backgrounds, values, views on handling disciplinary blind spots, and exposure to ethics in design. Chloé demonstrated the most knowledge about gendered and racial bias in design and how these can influence design ethics.

The inclusion of red flags in the study was intentional. This was to assess how participants would interact with them. The reason that cues pertaining to race and pain were included in the scenario is because research shows that social injustices are embedded in racially discriminatory algorithms (El-Azab & Nong, 2023). Furthermore, Black patients are believed to exaggerate their pain levels and should therefore not receive pain medication as readily as other patients (Vyas et al., 2020, p. 874, p. 879). Against the backdrop of such racism, what set Brandon apart from the other participants was his opposition to including race in the algorithm. In the interview, he elaborated on his principled stance, further justifying the need to go beyond generalizations about robotics engineers.

In terms of social positionality, understanding participants’ motivations that led them to their current studies are helpful markers of their underlying intentions in the field of robot design. Chloé and Rebecca pointed to the importance of ‘women in engineering’ events that inspired them to pursue their studies. Rebecca went on to describe the role of her father (an engineer), while Brandon reminisced about childhood experiences with science. Chloé was well-versed with ethical concerns about gendered and racial bias in design and how these can influence design ethics. There is no clear indication that the men who participated in the study have limited worldviews that impact their design. In fact, it was Brandon who was most vocal about excluding race from the algorithm. The evidence from the study reveals that participants defied the sentiment of ‘antisocial today and also always’; they are aware of social problems in design and seek collaboration for inclusive design. The issue that requires further research is if their studies prepare them sufficiently at an undergraduate level to consider ethical challenges in design.

All participants acknowledged that they have blind spots, and all participants valued collaboration in some form to deal with this problem. It was noticeable that two participants positioned the engineer as the central figure, while other participants valued collaboration as being between equal partners. This is connected to the strong sense of social justice that was conveyed by all participants. While there are glimpses of this originating in their course work, it seems that the main sources of social justice come from lived and social experiences. The implication of this is that engineering course work at the university, according to participants, has much room to either redesign the programme so that ethics and sociology-oriented courses can be included, or for these components in existing courses to receive greater weighting. If students believe that they can pass an engineering course without focusing on ethics, as Sanath pointed out, then this is a concern because – whether true or not – it signals a perception of design as the priority, and social responsibility as a secondary consideration. If ethics is to be included in a way that students appreciate, then this needs to be done *by design*.

Our investigation was limited to the use of one type of social robot due to its popularity in research and ease of programming. It is possible that programming a different social robot (e.g. in morphology, behaviour, programming language) could yield differing results. However, given the demonstrated promise of the NAO robot for clinical applications in research, we believe the robot is a suitable and relevant paradigm to probe links between engineering training and designer bias.

## Conclusion

This exploratory research has shown that undergraduate mechatronics and robotics students who participated in the study do not conform with the caricature of “antisocial engineers.” While they sometimes reproduced assumptions of engineering authority in design, they also expressed humility about disciplinary limits, an openness to collaboration, and even a willingness to challenge ethically questionable demands in the study prompt. For example, Brandon’s refusal to accept race as a category in design is evidence of ethical resistance.

Yet the findings also reveal a troubling disjuncture: social and ethical considerations are present in the engineering curriculum at the S.E Ontario university but empirical findings suggest that participants rarely felt it is framed as integral to their degree requirements. Students recognized ethics as important but their impressions also mirrored their framings of it as supplemental; something that is an afterthought in their courses, or to possibly be taken as an elective in the third or fourth year of study. This should be read not as student apathy, but, from a student perspective, a reflection of an institutional gap in engineering education at this university. If ethics is not integrated meaningfully into the curriculum at the undergraduate level, and instead remains as an insignificant add-on, then it is likely to inform student perceptions of its (in)significance.

Robots in healthcare are not neutral technologies because they reflect designer biases. As a result, they can contribute to the reproduction of systemic inequalities or to their disruption. Whether they exacerbate injustice or contribute to more equitable healthcare could depend, in part, on how engineering students are taught to think about their roles in patient care. Our data suggest that students are already self-reflective on matters of social consideration in robot design, but without curricular transformation, those personal values amongst study participants may not be engrained as core considerations in design.

For this reason, future research must not only examine engineering students’ ethical and social values in design but also interrogate engineering programs as socio-political sites where the value systems of future engineers are actively shaped. What might it mean to treat engineering education as a form of social justice training rather than as a pipeline to industry? How might ethics be embedded not as a sidebar but as a cornerstone of technical practice? It is our hope that future research will consider these questions.

## Data Availability

Not applicable.

## Appendix A

### Background

The robot here is called NAO. You have been tasked to program NAO to be used in the medical industry, such as in a hospital or clinic setting. NAOs have the same privileges as Registered Nurses (i.e., they can write prescriptions) and interact directly with patients to enable more prompt assistance and help solve backlogs at medical facilities. NAO will ask the patient questions, and then, at the end of the exchange, the NAO will decide whether to write a prescription or refer the patient to a doctor or nurse for further examination.

### The Task

A patient comes in with pain.

Your task is to get NAO to obtain a profile, do a questionnaire checkup with this patient, and then either state that it will write a prescription or refer the patient to a doctor or nurse for further examination. While completing your checkup, you need to retrieve enough information from the patient to assess 1) their level of pain, 2) their location of pain, 3) any recent medication, 4) their history of pain, 5) their race (as this might inform how much pain medication they receive), and any other factors that might be informative towards NAO’s final decision. You have a max of 1 hour for this task.

You may look at the examples of standard dialogues to help with programming NAO and the provided cheat sheet.

Please ask me if you have any questions or concerns about this task as you proceed.
